# Factors Affecting the Initiation of a Shared Decision Making Program in Obstetric Practices

**DOI:** 10.3390/healthcare9091217

**Published:** 2021-09-16

**Authors:** Deborah J. Bowen, Ann M. Nguyen, Cynthia LeRouge, Erin LePoire, Tao Sheng Kwan-Gett

**Affiliations:** 1 Department of Bioethics and Humanities, School of Medicine, University of Washington, 1959 NE Pacific St., Room A204, Box 357120, Seattle, WA 98195, USA; lepoire2@uw.edu; 2 Rutgers Center for State Health Policy, 112 Paterson St., 5th Floor, New Brunswick, NJ 08901, USA; anguyen@ifh.rutgers.edu; 3 Department of Information Systems & Business Analytics, College of Business, Florida International University, 11200 SW 8th St., R.B. 206B, Miami, FL 33199, USA; clerouge@fiu.edu; 4 Department of Health Systems and Population Health, School of Public Health, University of Washington, 3980 15th Ave NE, Fourth Floor, Box 351621, Seattle, WA 98195, USA; kwangett@uw.edu

**Keywords:** shared decision making, project initiation, patient decision aid, implementation science, healthcare project management

## Abstract

As healthcare systems progress toward initiatives that increase patient engagement, stakeholder hopes are that shared decision making (SDM) will become routine practice. Yet, there is limited empirical evidence to guide such SDM program implementations, particularly in obstetric practices. The first stage of any project implementation is the “initiation stage”, in which project leaders define a project’s purpose and stakeholders and structures are put in place to support the new initiative. Our study’s objective was to identify factors affecting the initiation stage of an SDM program implementation project for TOLAC, trial of labor after Cesarean. We conducted a multiple-case study of an SDM program implementation in three obstetric settings in Washington State. The research design and analysis were guided by implementation science frameworks and project management literature. Data sources included interviews with key informants from the State, SDM tool vendors, and three project sites, as well as implementation documents. The study results provide insight into how the identified project implementation factors provide an essential foundation for informing project planning, execution, and reflection/evaluation. In this study, the State’s decision aid certification program pressured the project sites to shape the project purpose and engage stakeholders that would meet immediate project requirements (specifically, state requirements). The study reveals that external demands may not be in perfect alignment with the internal necessities required for an SDM program’s long-term viability and sustainability. Findings may be used by implementers and researchers to model and strategize the early stages of SDM program implementation projects, particularly in the obstetric setting.

## 1. Introduction

Shared decision making is the process in which a provider and patient make a medical decision together—combining clinical evidence with the patient’s values and preferences [1]. One such decision is how to deliver a healthy baby after a previous Cesarean section. Generally, pregnant women who had a previous Cesarean section face a decision either to have another Cesarean section or to attempt to deliver vaginally (i.e., trial of labor after cesarean (TOLAC) (Abbreviations used in this paper: Trial of labor after cesarean section (TOLAC); shared decision making (SDM); Consolidated Criteria for Reporting Qualitative Research (COREQ))). However, there is a lack of clarity in clinical guidelines on TOLAC [2]. Providers and patients must thus be willing and able to discuss delivery options knowledgeably—that is, engage in shared decision making (SDM) [3].

Several structural elements facilitate SDM: patient decision aids, provider training, and a means to record the SDM process in patient records. An SDM program typically includes all of these tools [4]. Prior research has indicated that having a decision aid selected is beneficial to the patient because the use of such an aid improves patient knowledge [5], care efficiency, and care quality [6,7]. Despite these benefits, however, very few SDM tools have been implemented in healthcare systems [8]. We propose that this lack of uptake is due to an inattention to other structural elements regarding SDM implementation.

In 2010, the promotion of decision aid certification by the Patient Protection and Affordable Care Act spurred efforts to increase SDM across the U.S. [9]. In 2016, Washington State became the first to launch a state-wide SDM initiative, which included certification of decision aids for TOLAC and implementing an SDM program in obstetric settings [10]. There is limited guidance on how to implement an SDM program in obstetric settings successfully. The absence of strong guidance poses an opportunity to learn from Washington State’s SDM program implementation in obstetric settings.

This study focuses on the first stage of an implementation project, which the implementation literature calls the “initiation” stage. The initiation stage is when the purpose of implementation and the stakeholders involved are defined [11,12,13,14]. A preliminary review of the literature uncovered some existing research on project initiation in the healthcare context (e.g., ref. [15] related to telemedicine projects and [16] related to electronic health record projects). Little is known about obstetrics situations.

The objective of our study was to identify factors affecting the initiation stage of an SDM program implementation. We conducted a multiple-case study of the SDM program implementation for TOLAC decision making in Washington State. Our study is the first to apply implementation science frameworks to the context of SDM project initiation and the obstetric setting. Our research is also enhanced by a complex external environment, in which a decision aid was certified by the State. Implementers and researchers may use findings to plan for the implementation of SDM programs in obstetric settings.

### Conceptual Model

**Figure 1:**
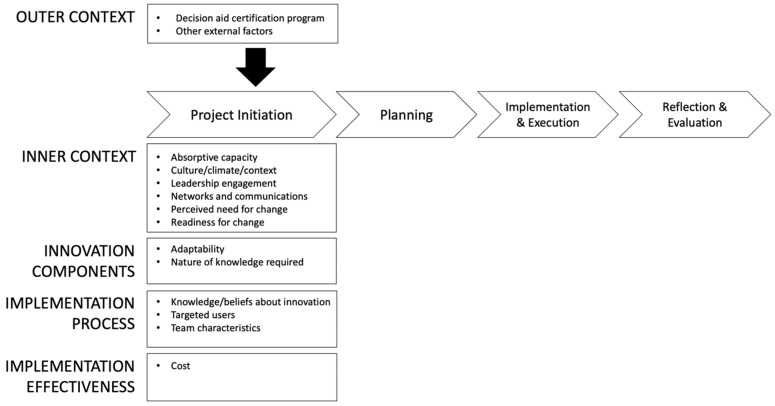
Conceptual model; note—Model draws upon [17,18,19].

We canvassed the implementation science literature for conceptual models that focused on innovation, as the innovation itself (SDM and decision aids) is pertinent to project initiation (i.e., project definition occurs during initiation). We identified three implementation science models [17,18,19] that conceptualized “innovation” and combined them [20] to create a conceptual model for our study. As shown in Figure 1, project initiation is conceptualized by four domains: inner context, innovation components, implementation process, and implementation effectiveness. These implementation models also suggest that project initiation is influenced by the outer context (i.e., the decision aid certification program, described more below, and other external factors). Per implementation literature [11], project initiation is followed by the stages of planning, implementation and execution, and, finally, reflection and evaluation. Our study focuses only on the project initiation stage.

## 2. Material and Methods

### 2.1. Design

We used a qualitative, multi-case study research design [21] consisting of three obstetric settings (i.e., project sites) participating in a state-sponsored SDM program implementation. Our study examines data from key informant interviews with multi-level stakeholders and implementation-related documents to identify factors affecting the initiation of the SDM program implementation in obstetric settings. This study was given an exempt determination by the Washington State Institutional Review Board [E-101916-A]. We used the COREQ guidelines to improve our presentation quality [22].

### 2.2. Study Setting

The project was part of a state initiative to increase the use of SDM in clinical practice. Central to the initiative was a decision aid certification program, in which an expert panel formally reviewed decision aids. More information about the certification program is available in a previous publication [23].

The State selected the three healthcare systems based on their high-volume obstetric programs. Table 1 gives the three systems’ profiles, herein referred to as Project Sites A, B, and C. There were two decision aid vendors involved in this project. One vendor’s decision aid was submitted to the certification program and formally certified. The other vendor’s decision aid was not submitted to the program. We refer to the two vendors as Certified Vendor and Non-Certified Vendor, respectively. The Certified Vendor also created the SDM concept training modules.

### 2.3. Data Sources

The primary data source was key informant interviews conducted before SDA program implementation with stakeholders from the State, vendors, and project sites. Key informants were selected via purposive sampling to represent the type of stakeholders involved in the implementation (i.e., executive sponsor, clinical champion, project manager/business analyst, IT analyst/interface engine programmer, provider, and subject matter expert consultant). We sent an email invitation to each organization’s point of contact with the research prospectus and a list of requested roles. All points of contact agreed to participate and connected us to individuals in the roles as relevant. Interviews were conducted until we reached thematic saturation [24], after 15 interviews. We continued to a final sample of 18 participants. See Appendix A for descriptions of the roles and distribution of participants across the roles. The semi-structured interview protocol was guided by our conceptual model and adapted to the stakeholder type. The lead investigator (CL, female) and an experienced research assistant (AN, female) conducted the interviews. Interviews were 45–60 min, conducted by phone, audio-recorded with permission, and transcribed. Recordings and transcripts were stored on a secure server accessible only to the research team.

Secondary data sources were documents related to the SDM tools (i.e., the decision aids, training materials, and EHR documentation protocol); implementation plans provided by the vendor; and project management plans, training modules, and EHR-related content provided by the project sites.

### 2.4. Data Management and Analyses

Data sources were analyzed and triangulated for key themes regarding organization factors affecting the initiation of an SDM implementation. We used the conceptual model as our coding schema, applying procedures described by [25] and investigator triangulation [20]. Two team members (AN, SS) coded each transcript/document independently and met periodically to calibrate during the coding process. Coding consisted of deductive assignment to the coding schema and inductive addition of detailed child codes. A third team member (CL) reviewed all coded text to assess the accuracy of the assigned codes. Consistent with processes in other qualitative studies [26], data analysis was complete when the coding team reached a consensus that all quotes were coded appropriately, and the resulting themes were stable. Qualitative data reporting was guided by the Consolidated Criteria for Reporting Qualitative Research (COREQ) [22]. We used Dedoose© software for all qualitative data management and analysis [27].

## 3. Results

The initiation stage of a project is when the purpose of the implementation and the stakeholders involved are defined. Overall, participants believed that project initiation was critical to ensuring the higher success of the SDM program implementation, particularly for obtaining the resources needed and getting the right stakeholders at the table. Each of the three project sites was affected by their inner context, innovation components, implementation process, and implementation effectiveness during project initiation, which shaped how the project was uniquely defined in each site and which stakeholders bought into the project. Project initiation was made even more complicated by the decision aid certification program and related external factors. These factors pressured project leaders to define the project purpose and identify stakeholders to meet immediate project requirements (i.e., state requirements) versus focusing on what may be necessary for the SDM program’s long-term viability and sustainability. Below, we describe these factors aligning with the initiation domains. In general, the sites provided assenting views except where otherwise indicated. Table 2 gives an evidence trace table for each domain and corresponding constructs.

### 3.1. Inner Context

Absorptive Capacity. During the initiation stage, all project sites were initially enthusiastic about the SDM program implementation and their abilities to “absorb” the program into routine operations. However, their enthusiasm began to drain as they realized the number of competing priorities that would lead to delays in project planning. Project site stakeholders reported feeling frustrated because the delays were due to “*factors out of our control*” (Project Site A). For example, Project Site C was undergoing a system-wide EHR upgrade, which reduced the stakeholders’ capacity to be involved (e.g., the IT analyst would not have the bandwidth to support the SDM program implementation until the EHR upgrade was complete).

Culture/Climate/Context. In defining the implementation’s purpose, TOLAC was chosen as the medical decision of initial focus for the SDM program because it was a medical topic with a receptive culture and showed promise from clinical success stories. Leaders across our interview groups felt that SDM about TOLAC could facilitate the organic adoption of SDM in other areas.

Leadership Engagement. Participants agreed that leadership engagement at all healthcare system levels was imperative in defining the purpose of the implementation and identifying the right stakeholders to be involved. In initiating this project, the State shared that they engaged leadership from the certified vendor, including their clinical champions, and asked them to meet with leadership at the healthcare systems to which the three obstetric sites belonged. In other words, the State recognized the importance of involving high-level leadership in early discussions about the implementation, alongside the administration at the local obstetric clinics. The certified vendor held early group meetings with the healthcare system leadership and potential implementation team members from the obstetric project sites for all three project sites. The purpose of these meetings was to build consensus on the project definition and the stakeholders to engage in project activities relative to each site’s goals.

Network and Communications. All participants noted that an SDM community would be valuable for defining the purpose of SDM program implementations and defining who should be involved. During project initiation, however, even though all three project sites were obstetric settings, they worked internally and did not communicate directly with one another about the specifics of their implementations. All three, however, involved the implementation vendor during project initiation. The vendor created a roadmap for each to use in their project planning.

Perceived Need for Change. Participants from the State and the vendors noted that the implementation’s purpose was shaped by a nationwide need for change towards SDM. Specifically, there was a need to engage patients better and reduce medical variation to reduce costs. They further cited that TOLAC had been identified as a priority area due to high variation across the nation, including in Washington State. Thus, the State was strongly motivated about the SDM program implementation and engaged directly with the project sites and the certified vendor during project initiation.

Readiness for Change. Project initiation was shaped by the readiness for change of all organizations involved. The State reported that they were primed for change and well-positioned to launch the program; they also had a local role model clinical champion with 3.5 years of SDM experience serving as their consultant. The State remarked that at the heart of the many obstacles to SDM program adoption is a “chicken and egg” (State) phenomenon. For healthcare systems to adopt SDM widely, SDM proponents need to demonstrate that SDM works; yet, to accrue evidence of SDM’s value, SDM needs to be widely adopted. The hope was that the project program would “plant the seeds” (State) of SDM to grow across the State, starting with the obstetric setting. For many of the project sites, however, readiness for change was mixed. The State contractually bound the projects to participate in the SDM program implementation; therefore, conversations about program implementation began as a means to comply with state requirements. However, all sites emphasized that they believed SDM was the right thing to do for patients and families and appreciated the external motivation to turn their beliefs into action.

### 3.2. Innovation Components

Adaptability. Participants noted that the SDM program’s adaptability affected the project definition and who would need to be involved in the project. At the forefront, participants debated which modality (i.e., paper, electronic, or class) of the SDM TOLAC decision aid to implement. Each modality had different implications for the resources and stakeholders needed. Participants noted that the paper and classroom options would require less upfront time and resources for implementation because they did not require EHR system modification. All sites reported that they preferred electronic decision aids and hoped to one day move in that direction to meet the needs of younger patients. Secondarily, participants noted that TOLAC, while a relatively straightforward decision (because there are only two choices—vaginal delivery or Cesarean section), is complicated because it does not need to be fixed or made at a single timepoint. Participants remarked that stakeholders needed to understand that during project initiation, the program could be adapted to other maternal health decisions beyond TOLAC. A TOLAC focus alone may not be a sufficient purpose for an obstetrics SDM program implementation.

Nature of Knowledge Required. Relatedly, participants considered the nature of knowledge required to implement an SDM program successfully. The State and both vendors stressed that, for the SDM program to be successful, everyone at the obstetric clinics needed to understand the SDM as a “bundle” (Certified Vendor), including concepts of SDM and how it differs from patient education. In other words, the State and vendors made sure to define the SDM program implementation by its multiple tools (e.g., TOLAC decision aid, provider SDM training, and process for recording SDM in patient records), and not just by the decision aid alone.

### 3.3. Implementation Process

Knowledge/Beliefs about Innovation. Participants reported that the SDM program initiation needed to account for providers’ knowledge and beliefs about SDM. In our interviews, we found that there were indeed differences in providers’ knowledge and opinions of SDM, mainly how SDM was related to patient education and informed consent. Some believed the terms to be relatively interchangeable, and therefore SDM was not a new concept. Others noted a clear difference, emphasizing that SDM is a more prescriptive process than patient education that requires more documentation. Given this variation in providers’ knowledge and opinions, participants recommended that the project initiation be deliberately explicit that one purpose of the project was to educate providers at obstetric clinics on core concepts of SDM—essentially, that the SDM purpose was beyond the context of TOLAC.

Targeted Users. The targeted users also defined the project. The direct users of decision aids are providers and patients. However, participants reported that during project initiation, they needed to engage “indirect users.” For example, at Project Site C, the implementation team decided to offer SDM training to all the staff in their clinic, including the non-clinical front desk staff and a system-level maternal health workgroup, which included providers and staff outside of their clinic. Regarding patient users, participants noted that 100% patient adoption was a “dream” (Project Site B), citing language and literacy barriers and that it was more realistic to aim for 75% patient adoption. In other words, the purpose of the SDM program was to optimize the adoption of the program in obstetric settings across direct and indirect users while recognizing that 100% adoption was unrealistic.

Team Characteristics. The assembly of the SDM program implementation teams was dependent on how the program was initiated. Participants reported that it was essential to identify a clinical champion during project initiation who would “keep the fire alive” (Project Site A), particularly during initiation delays. The clinical champion could also help obtain senior leadership support to allocate resources to the project, allowing the project to advance into the planning stage. The two project sites that cited having strong clinical champions and leadership support during project initiation ended up having larger implementation teams and more internal financial backing.

### 3.4. Implementation Effectiveness

Cost. As participants noted, the cost is rarely absent from the discussion of any project initiation. Implementing an SDM project is an expensive undertaking. Hence, participants relayed that healthcare system stakeholders need to be convinced that SDM adds value to providers and patients before making such an investment. Participants indicated that demonstrating the return-on-investment of SDM is difficult, especially during the initiation of a novel project like this, which has limited evidence on cost savings. They noted that healthcare systems operating with thin margins are unlikely to have extra money to invest in decision aids independently. In our case study, the State was sponsoring the program implementation, and it was imperative to make this clear at the onset. However, the costs needed to sustain the program beyond this initial implementation were unclear and affected the initial project site buy-in.

### 3.5. Outer Context

Decision Aid Certification Program and Other External Factors. Interwoven with the factors of the inner context, innovation components, implementation process, and implementation effectiveness was the decision aid certification program (and related other external factors). Participants described the decision aid certification program as both a motivation and concern for project initiation. The certification of TOLAC decision aids was welcomed by the vendors, who appreciated additional validation that their decision aids were balanced and unbiased. The project site participants also responded to the external push to implement something they believed was valuable to patients and families (see Readiness for Change section). Thus, the certification program provided a purpose—an impetus—for the State, vendors, and program sites to implement the SDM program: to assess the role of TOLAC decision aid certification in the adoption of an SDM program. Certification of TOLAC decision aids also has legal implications. The State intended to provide additional legal protections and seek reduced malpractice insurance rates if SDM was documented in a patient’s record. Some provider participants had noted that legal protections were critical to include in the project definition because a failed TOLAC could lead to lawsuits. The providers noted that some obstetric gynecologists lean away from TOLAC to avoid potential poor outcomes for their patients and their patients’ babies, in which they would have to do an emergency Cesarean section. This component affected the types of stakeholders (e.g., senior leadership and legal team) that needed to be engaged and consequentially sold on the project during project initiation.

## 4. Discussion

Our multiple-case study identified factors affecting the initiation of an SDM program implementation in three obstetric settings. Our findings can guide future SDM program implementations and research in this area through the following study implications. First, we found that participants believed that deliberate attention to project initiation resulting in viable, propagated project conceptualization is critical to SDM program implementation success. Successful project initiation results in a shared understanding of the project’s purpose and the identification of stakeholders that need to be involved [11]. Project initiation has been described in other settings (e.g., for telemedicine implementation [15]) as essential for project buy-in. Initial buy-in and shared understanding set a strong foundation to support successful project planning, implementation/execution, and finally, reflection/evaluation. Per SDM experts Légaré and Witteman, SDM can only be integrated into routine care delivery when the components of SDM are organized to align with the principles of patient engagement [28]. It is during the project initiation stage that centering on patient engagement can happen, motivating stakeholder buy-in and eventual adoption.

Second, our findings reveal that, during the project initiation stage, multiple factors of the inner context, innovation components, implementation process, implementation effectiveness, and outer context shaped how project leaders uniquely defined the project at each site and which stakeholders were actively engaged in the project. In this study, project initiation was complicated by the State’s decision aid certification program and related external factors, which pressured the project sites to shape the project purpose and engage stakeholders that would meet immediate project requirements (specifically, state requirements). Our study reveals that external demands may not be in perfect alignment with the internal necessities required for an SDM program’s long-term viability and sustainability. Further, stakeholders’ negative beliefs about the value propositions of SDM and the TOLAC medical decision can de-rail a project during initiation. In this case study, the State’s certification of a TOLAC decision aid may have been critical to moving the SDM initiative forward by signaling to the project sites that SDM is important. As indicated by some participants in our study, the application of SDM tools to support the medical decision of TOLAC alone was not seen as a sufficient purpose for an SDM program implementation. During initiation, it is vital to be clear in the project purpose (e.g., its mission statement) that the overarching goal is to integrate SDM into routine practice, with TOLAC as the first of many medical decisions within a comprehensive SDM program. The State’s active engagement and interest may help stakeholders see the big picture context and become actively engaged in the project.

Third, during project initiation, it is important for implementers to recognize that SDM programs are more than choosing and obtaining a decisional tool [29]. SDM training and EHR documentation are additional components of the SDM toolkit that can impact the implementation purpose and types of stakeholders who need to be involved because of their implications on cost and timelines. As SDM programs are indeed a bundle of components (directed at process, tools, and expanding mindsets) and not just a standalone decision aid, it is crucial during initiation to be clear to stakeholders about the challenges involved in measuring return-on-investment. These challenges are due mainly to an SDA program’s multiple components and lack of defined measures [30].

## 5. Limitations

We made many decisions for this project that limited the generalizability. First, this is a study focused on three clinical settings, and other settings may have different issues and considerations. Second, the sample size was small, and not sampled in a rigorous way. Therefore, we may have engaged bias in the sampling process. Third, there are no data presented to indicate that clinical care has changed as a result of implementing this shared decision process. We would need to follow these patients and compare them with patients who do not participate in SDM during their delivery choices. For these reasons, we consider this to be an initial study of SDM and will consider a larger, more fully powered study of multiple health care systems to fully understand this phenomenon.

## 6. Conclusions

Our findings contribute to the field by providing insights for advancing SDM implementation success by focusing on the initiation stage of an SDM project implementation, which provides an important foundation to informing project planning, executing, and reflecting/evaluating. Many of our key takeaways align with an earlier systematic review on providers’ perspectives of the barriers and facilitators to SDM implementation [31]. This study adds to the literature on the perspectives of multi-level stakeholders and teases out factors for consideration during project initiation. We reveal considerations and best practices regarding SDM project implementation during the initiation stage by coupling informants of multiple roles with our integrative conceptual framework.

## Figures and Tables

**Table 1:** Profiles of the project sites.

	Project Site A	Project Site B	Project Site C
**Healthcare System Type**	Academic, Voluntary Nonprofit	Governmental Hospital District	Voluntary Nonprofit
**Location**	Urban	Urban	Urban
**FQHC Status**	No	No	No
**# of Obstetric Clinics in Project**	2	5	1
**# of Clinicians in Project**	17 (9 OBGYNs, 8 midwives)	40 (all OBGYNs)	3 (2 OBGYNs, 1 midwife)
**Decision Aid Vendor**	Non-Certified Vendor	Non-Certified Vendor	Certified Vendor
**Decision Aid Modality**	Paper	In-Person Class	Paper
**EHR System**	Epic©	Different system at each clinic	Epic©

Note. EHR = electronic health record; FQHC = federally qualified health center; OBGYN = obstretic-gynecologist; SDM = shared decision making.

**Table 2:** Evidence trace table.

Theme	Source	Representative Supporting Evidence (Quote or Supporting Document Comment)
**Inner Context**		
**Absorptive Capacity**	Interview	“We were just coming out of a huge, Epic© upgrade, and IT was quite busy. Our physicians were happy to at least have something that they can start working on [the paper version of the decision aid]. We can potentially see an electronic decision aid implemented some time down the line or maybe next year once we have our resources back in place.” (Project Site C)
	EHR documentation protocol	Early versions of some EHR documentation protocols were for an electronic version of the decision aid but were then updated to documentation of a paper-based or classroom version.
**Culture/Climate/Context**	Interview	“[There is] a very activated obstetrical community around obstetrical quality improvement, especially with respect to active management of labor.” (State)
**Leadership Engagement**	Interview	“Part of the initial conversations with the [State] was alongside our Director for the Clinical Integrated Network. Both of them were on initial conversations. and we felt that it would be great for [Health System] to start this project.” (Project Site C)
	Project management plans provided by project sites	Project management plans noted who was involved in the initial meetings from each project site and healthcare system.
**Network and Communications**	Interview	“The willingness to share [information] with each other, even amongst organizations that are competing with each other, is probably stronger than average.” (Certified Vendor)
	Implementation plans provided by the vendor	Each project site received an implementation plan (i.e., roadmap) from the vendor, and the roadmaps had similar components.
**Perceived Need for Change**	Interview	“If [the patient] has all the information, it’s possible that they would pick the less expensive option.” (Vendor)
**Readiness for Change**	Interview	“SDM is the right thing to do for our patients and families.” (Project Site A)
**Innovation Components**		
**Adaptability**	Interview	“The IT build is one of our major time obstacles. We don’t have an IT department that’s going to prioritize this as number one, and so we needed something fairly simple for them to be able to get it done in less than a year, really.” (Project Site A)
	Training modules provided by project sites	Training modules indicated that the decision aid could be presented to the patient at several possible timepoints.
**Nature of Knowledge Required**	Interview	“The VBAC [Vaginal Birth After Cesarean] booklet is part of just a bigger education campaign.” (Non-Certified Vendor)
	Implementation plans provided by the vendor	The implementation plans laid out multiple components of the SDM program, including implementation of the TOLAC decision aid, provider SDM training, and process for recording SDM in patient records.
**Implementation Process**		
**Knowledge/Beliefs about Innovation**	Interview	“It used to be called informed consent. To tell you the truth, most physicians find it kind of amusing that somebody decided to rename it, which is what they do with everything every few years. In theory, it’s different, but it’s not in practice.” (Project Site B)
	Interview	“To me, shared decision making is following a very prescriptive process that documents key milestones in the conversation that unravels with the patient and the family, being able to point them to the risks and the benefits of a chosen treatment option, being able to document that, being able to then arrive at a decision and to measure its impact and evaluation.” (Project Site A)
**Targeted Users**	Interview	“Honestly, I think if we can get 60 to 70% [of eligible patients through the class], that would be a huge win. You just can’t make everybody can go to the class. There are people who can’t make it or are not going to. I don’t think that will ever have 100%. That’s a dream. I would be really happy if we got 60 to 70. I feel like 75% would be an absolutely stellar result.” (Project Site B)
	Training materials	Training materials described the types of users.
**Team Characteristics**	Interview	“The non-physician leaders [at Project Site] were pretty much on board, ready to go, but there were some physician doubts on it about whether or not this was worthwhile, how exactly it would work.” (Certified Vendor)
	Project management plans provided by project sites	Project management plans listed the team members.
**Implementation Effectiveness**		
**Cost**	Interview	“My sense is that organizations are doing as much as they can to cut cost, and I think SDM is one because, it’s really hard to show the return-on-investment of a program like this. I imagine that the growth in the momentum will continue to be slow unfortunately.” (State)
**Outer Context**		
**Decision Aid Certification Program and Other External Factors**	Interview	“I think in the end that external environmental driver that has in Washington State that this reduces your medical malpractice liability is the big opportunity for us push things in the right direction. And eventually we’re going to go there.” (Certified Vendor)

## Data Availability

Data will be made available to investigators upon reasonalble request.

## Appendix A

**Table A1:** Interview participant roles.

Role	Responsibilities Related to SDM Project	Number of Participants (*N* = 18)					
		Case Study Site A (*n* = 4)	Case Study Site B (*n* = 3)	Case Study Site C (*n* = 2)	State Government Sponsor (*n* = 4)	Certified Vendor (*n* = 4)	Non-Certified Vendor (*n* = 1)
**Executive sponsor**	Secures spending and resource requirements Acts as vocal and visible business champion Approves scope changes Ensures continuity of sponsorship	1	0	0	1	0	0
**Clinical champion**	Educate peers on importance and value of project Advise on product configuration options to align with clinical workflow	1	2	1	1	1	0
**Project manager/business analyst**	Primary SDM vendor contact Ensures overall project success Helps schedule regular check-in calls with project team Communicates barriers to executive team Coordinates training and product configuration efforts Ensures weekly action items are tracked and completed	1	1	1	1	2	1
**IT analyst/interface engine programmer**	Configures EHR interface to allow for ordering Configures EHR interface to present completion status and patient response back into record Configures client interface engine based on technical requirements Communicates with interface engine vendor (if necessary)	1	0	0	0	0	0
**Provider**	Will deliver SDM at the site	0	0	0	0	0	0
**Subject matter expert consultant**	Advises implementation team on importance and value of project Advises on product configuration options	0	0	0	1	1	0

Note. EHR = electronic health system; IT = information technology; IP = Internet protocol; SDM = shared decision making; VPN = virtual private network.
